# Supplementary material for: Clinical factors associated with microstructural connectome related brain dysmaturation in term neonates with congenital heart disease
Source: Front Neurosci. 2022 Nov 18;16:952355. doi: 10.3389/fnins.2022.952355 (PMC9717392; doi:10.3389/fnins.2022.952355)
Supplement: Supplementary file 1 [file Data_Sheet_1.PDF]

Supplemental Table 1: Anatomic Labels for the 90 AAL Template Nodal Structures Used in the Nodal Connectome Analysis

Node	Abbreviation	Node	Abbreviation	Node	Abbreviation
Amygdala, Left	AMYG.L	Superior Frontal Gyrus, Medial, Right	SFGmed.R	Superior Parietal Gyrus, Left	SPG.L
Amygdala, Right	AMYG.R	Superior Frontal Gyrus, Orbital Part, Left	ORBsup.L	Superior Parietal Gyrus, Right	SPG.R
Angular Gyrus, Left	ANG.L	Superior Frontal Gyrus, Orbital Part, Right	ORBsup.R	Postcentral Gyrus, Left	PoCG.L
Angular Gyrus, Right	ANG.R	Superior Frontal Gyrus, Dorsolateral, Right	SFGdor.R	Postcentral Gyrus, Right	PoCG.R
Calcarine Fissure, Left	CAL.L	Fusiform Gyrus, Left	FFG.L	Precentral Gyrus, Left	PreCG.L
Calcarine Fissure, Right	CAL.R	Fusiform Gyrus, Right	FFG.R	Precentral Gyrus, Right	PreCG.R
Caudate Nucleus, Left	CAU.L	Heschl Gyrus, Left	HES.L	Precuneus, Left	PCUN.L
Caudate Nucleus, Right	CAU.R	Heschl Gyrus, Right	HES.R	Precuneus, Right	PCUN.R
Anterior Cingulate Gyrus, Left	ACG.L	Hippocampus, Left	HIP.L	Putamen, Left	PUT.L
Anterior Cingulate Gyrus, Right	ACG.R	Hippocampus, Right	HIP.R	Putamen, Right	PUT.R
Middle Cingulate Gyrus, Left	MCG.L	Insula, Left	INS.L	Gyrus Rectus, Left	REC.L
Median Cingulate Gyrus, Right	MCG.R	Insula, Right	INS.R	Gyrus, Rectus, Right	REC.R
Posterior Cingulate Gyrus, Left	PCG.L	Lingual Gyrus, Left	LING.L	Rolandic Operculum, Left	ROL.L
Posterior Cingulate Gyrus, Right	PCG.R	Lingual Gyrus, Right	LING.R	Rolandic Operculum, Right	ROL.R
Cuneus, Left	CUN.L	Inferior Occipital Gyrus, Left	IOG.L	Supplementary Motor Area, Left	SMA.L
Cuneus, Right	CUN.R	Inferior Occipital Gyrus, Right	IOG.R	Supplementary Motor Area, Right	SMA.R
Inferior Frontal Gyrus, Opercular Part, Left	IFGoperc.L	Middle Occipital Gyrus, Left	MOG.L	SupraMarginal Gyrus, Left	SMG.L
Inferior Frontal Gyrus, Opercular Part, Right	IFGoperc.R	Middle Occipital Gyrus, Right	MOG.R	SupraMarginal Gyrus, Right	SMG.R
Inferior Frontal Gyrus, Orbital Part, Left	ORBinf.L	Superior Occipital Gyrus, Left	SOG.L	Inferior Temporal Gyrus, Left	ITG.L
Inferior Frontal Gyrus, Orbital Part, Right	ORBinf.R	Superior Occipital Gyrus, Right	SOG.R	Inferior Temporal Gyrus, Right	ITG.R
Inferior Frontal Gyrus, Pars Triangularis, Left	IFGtriang.L	Olfactory Cortex, Left	OLF.L	Middle Temporal Gyrus, Left	MTG.L
Inferior Frontal Gyrus, Pars Triangularis, Right	IFGtriang.R	Olfactory Cortex, Right	OLF.R	Middle Temporal Gyrus, Right	MTG.R
Superior Frontal Gyrus, Medial Orbital, Left	ORBsupmed.L	Pallidum, Left	PAL.L	Temporal Pole: Middle Temporal Gyrus, Left	TPOmid.L
Superior Frontal Gyrus, Medial Orbital, Right	ORBsupmed.R	Pallidum, Right	PAL.R	Temporal Pole: Middle Temporal Gyrus, Right	TPOmid.R
Middle Frontal Gyrus, Left	MFG.L	Paracentral Lobule, Left	PCL.L	Temporal Pole: Superior Temporal Gyrus, Left	TPOsup.L

Middle Frontal Gyrus, Right	MFG.R	Paracentral Lobule, Right	PCL.R	Temporal Pole: Superior Temporal Gyrus, Right	TPOsup.R
Middle Frontal Gyrus, Orbital Part, Left	ORBmid.L	ParaHippocampal Gyrus, Left	PHG.L	Superior Temporal Gyrus, Left	STG.L
Middle Frontal Gyrus, Orbital Part, Right	ORBmid.R	ParaHippocampal Gyrus, Right	PHG.R	Superior Temporal Gyrus, Right	STG.R
Superior Frontal Gyrus, Dorsolateral, Left	SFGdor.L	Inferior Parietal, Left	IPL.L	Thalamus, Left	THA.L
Superior Frontal Gyrus, Medial, Left	SFGmed.L	Inferior Parietal, Right	IPL.R	Thalamus, Right	THA.R

Supplemental Table 2(A). Correlation between Clinical Risk Factors and Seed-Based Tractography Measurements: Innate Factors and Cardiac Lesions

			Fractional Anisotropy			Radial Diffusivity			Axial Diffusivity		
**Independent**	Dependent										
**Clinical factors**	Tract	N used	FDR p-value	estimate	direction	FDR p-value	estimate	direction	FDR p-value	estimate	direction
**Birth weight**	Corpus Callosum Genu	67	0.8602	-7.60E-06	-	**0.041**	5.55E-05	+	**0.0557**	5.25E-05	+
	Corpus Callosum Body	67	0.3729	-1.30E-05	-	**0.0066**	9.28E-05	+	**0.0182**	8.73E-05	+
	Corpus Callosum Splenium	67	0.48	-1.70E-05	-	**0.0176**	9.63E-05	+	0.0908	7.52E-05	+
	Cortical Spinal Tract Left	63	0.8926	-1.50E-06	-	0.1017	7.77E-05	+	**0.0557**	0.000105	+
	Cortical Spinal Tract Right	64	0.8926	-2.20E-06	-	0.1008	8.63E-05	+	**0.0557**	0.000112	+
	Fronto-occipital Fasciculus Left	59	0.8926	-2.70E-06	-	0.191	3.62E-05	+	0.2057	3.81E-05	+
	Fronto-occipital Fasciculus Right	57	0.8926	1.01E-06	+	0.9824	6.29E-07	+	0.8984	3.61E-06	+
	Inferior Longitudinal Fasciculus Left	61	0.8926	1.73E-06	+	0.2988	3.51E-05	+	0.2073	4.70E-05	+
	Inferior Longitudinal Fasciculus Right	60	0.3729	-1.20E-05	-	0.1008	6.62E-05	+	0.2514	4.51E-05	+
	Superior Longitudinal Fasciculus Left	65	0.8926	-4.50E-06	-	**0.0187**	0.000101	+	**0.0182**	0.000119	+
	Superior Longitudinal Fasciculus Right	63	0.8926	-3.60E-06	-	0.1198	5.06E-05	+	0.0799	5.45E-05	+
**Birth weight, percentile**	Corpus Callosum Genu	67	0.4437	-0.00017	-	**0.0055**	0.001263	+	**0.0072**	0.001262	+
	Corpus Callosum Body	67	0.4437	-0.00022	-	**0.0022**	0.001723	+	**0.0066**	0.001722	+
	Corpus Callosum Splenium	67	0.4437	-0.00026	-	**0.0055**	0.001769	+	**0.0327**	0.001571	+
	Cortical Spinal Tract Left	63	0.9589	8.88E-06	+	0.2109	0.000916	+	0.1039	0.001349	+
	Cortical Spinal Tract Right	64	0.8817	-6.00E-05	-	**0.02**	0.001845	+	**0.0114**	0.002367	+
	Fronto-occipital Fasciculus Left	59	0.6474	-9.00E-05	-	**0.0426**	0.000937	+	**0.0327**	0.001016	+
	Fronto-occipital Fasciculus Right	57	0.8817	-4.60E-05	-	0.244	0.000566	+	0.1528	0.000688	+
	Inferior Longitudinal Fasciculus Left	61	0.9224	-2.60E-05	-	0.1223	0.000874	+	0.0886	0.001054	+
	Inferior Longitudinal Fasciculus Right	60	0.4437	-0.0002	-	**0.0426**	0.001243	+	0.1097	0.001028	+
	Superior Longitudinal Fasciculus Left	65	0.4437	-0.00013	-	**0.02**	0.001667	+	**0.0157**	0.001869	+

	Superior Longitudinal Fasciculus Right	63	0.4437	-0.00015	-	**0.0297**	0.001149	+	**0.0207**	0.001134	+
**Head circumference**	Corpus Callosum Genu	66	0.7138	-0.0008	-	0.6204	0.004624	+	0.5025	0.004974	+
	Corpus Callosum Body	66	0.587	-0.00135	-	0.6204	0.006139	+	0.5025	0.005072	+
	Corpus Callosum Splenium	66	0.7138	-0.00062	-	0.6204	0.005321	+	0.5025	0.006899	+
	Cortical Spinal Tract Left	62	0.587	-0.00215	-	0.6204	0.004631	+	0.8263	0.001507	+
	Cortical Spinal Tract Right	64	0.7138	-0.00127	-	0.6204	0.004117	+	0.6982	0.003366	+
	Fronto-occipital Fasciculus Left	59	0.7138	0.000545	+	0.6204	0.003382	+	0.5025	0.006741	+
	Fronto-occipital Fasciculus Right	57	0.7138	0.000965	+	0.9145	-0.00053	-	0.6539	0.003041	+
	Inferior Longitudinal Fasciculus Left	61	0.7138	0.001121	+	0.7084	0.002575	+	0.5025	0.007747	+
	Inferior Longitudinal Fasciculus Right	60	0.7138	-0.00044	-	0.6204	0.004291	+	0.5833	0.00548	+
	Superior Longitudinal Fasciculus Left	64	0.587	-0.00149	-	0.6204	0.008259	+	0.5025	0.00755	+
	Superior Longitudinal Fasciculus Right	62	0.7138	-0.00064	-	0.6204	0.003002	+	0.5833	0.003	+
**Head circumference, percentile**	Corpus Callosum Genu	66	0.8518	-6.30E-05	-	**0.0147**	0.001123	+	**0.0017**	0.00137	+
	Corpus Callosum Body	66	0.8518	-0.00012	-	**0.0147**	0.001366	+	**0.0077**	0.001499	+
	Corpus Callosum Splenium	66	0.8518	-6.70E-05	-	**0.0198**	0.001459	+	**0.0132**	0.001817	+
	Cortical Spinal Tract Left	62	0.9212	1.64E-05	+	0.2704	0.000832	+	0.123	0.00121	+
	Cortical Spinal Tract Right	64	0.8518	6.00E-05	+	0.4325	0.000539	+	0.2099	0.000976	+
	Fronto-occipital Fasciculus Left	59	0.8518	-4.60E-05	-	**0.0147**	0.001167	+	**0.0017**	0.001493	+
	Fronto-occipital Fasciculus Right	57	0.8518	3.48E-05	+	0.2866	0.000517	+	0.0573	0.000918	+
	Inferior Longitudinal Fasciculus Left	61	0.8518	0.000117	+	0.1702	0.000829	+	**0.0132**	0.001463	+
	Inferior Longitudinal Fasciculus Right	60	0.8518	-7.90E-05	-	0.1702	0.000882	+	0.1014	0.001021	+
	Superior Longitudinal Fasciculus Left	64	0.8518	-6.40E-05	-	0.0537	0.001368	+	**0.0257**	0.001606	+
	Superior Longitudinal Fasciculus Right	62	0.8518	4.03E-05	+	0.2552	0.000597	+	**0.0478**	0.000893	+
**Birth length**	Corpus Callosum Genu	63	0.7563	-0.0005	-	0.5731	0.001694	+	0.5909	0.001399	+
	Corpus Callosum Body	63	0.7563	-0.00029	-	0.5731	0.002269	+	0.5909	0.002744	+

	Corpus Callosum Splenium	63	0.7563	-0.00053	-	0.5731	0.002887	+	0.5909	0.003197	+
	Cortical Spinal Tract Left	59	0.7563	-0.001	-	0.9378	0.00026	+	0.6798	-0.00155	-
	Cortical Spinal Tract Right	61	0.7563	-0.00038	-	0.5731	0.00248	+	0.5909	0.00298	+
	Fronto-occipital Fasciculus Left	56	0.7563	0.000302	+	0.9378	0.000396	+	0.643	0.001308	+
	Fronto-occipital Fasciculus Right	54	0.7563	0.000482	+	0.9378	0.000221	+	0.5909	0.001812	+
	Inferior Longitudinal Fasciculus Left	58	0.7563	0.000248	+	0.5731	0.002413	+	0.5909	0.004154	+
	Inferior Longitudinal Fasciculus Right	57	0.9615	2.90E-05	+	0.5731	0.002537	+	0.5909	0.003782	+
	Superior Longitudinal Fasciculus Left	61	0.451	-0.00081	-	0.5731	0.004032	+	0.5909	0.003659	+
	Superior Longitudinal Fasciculus Right	59	0.451	-0.00092	-	0.5731	0.003003	+	0.5909	0.002043	+
**Birth length, percentile**	Corpus Callosum Genu	63	0.7599	-0.00013	-	**0.0407**	0.000844	+	0.0675	0.000775	+
	Corpus Callosum Body	63	0.8495	-3.00E-05	-	**0.0407**	0.001073	+	**0.0226**	0.001424	+
	Corpus Callosum Splenium	63	0.7599	-0.00015	-	**0.0407**	0.001525	+	**0.0315**	0.001662	+
	Cortical Spinal Tract Left	59	0.7599	-0.00011	-	0.506	0.000463	+	0.4902	0.000541	+
	Cortical Spinal Tract Right	61	0.9831	3.39E-06	+	0.0694	0.001176	+	**0.0315**	0.001757	+
	Fronto-occipital Fasciculus Left	56	0.7599	-5.00E-05	-	0.1129	0.000691	+	0.1283	0.000701	+
	Fronto-occipital Fasciculus Right	54	0.7599	7.30E-05	+	0.3509	0.00047	+	0.1067	0.000831	+
	Inferior Longitudinal Fasciculus Left	58	0.7599	6.02E-05	+	**0.0407**	0.001241	+	**0.0187**	0.001727	+
	Inferior Longitudinal Fasciculus Right	57	0.7599	-7.80E-05	-	**0.0407**	0.001324	+	**0.0315**	0.001533	+
	Superior Longitudinal Fasciculus Left	61	0.7599	-8.10E-05	-	**0.0418**	0.001316	+	**0.033**	0.001549	+
	Superior Longitudinal Fasciculus Right	59	0.4697	-0.00021	-	**0.0407**	0.001104	+	**0.0352**	0.000935	+
**APGAR, 1 minute**	Corpus Callosum Genu	61	0.1232	0.005087	+	0.0693	-0.01546	-	0.3914	-0.00679	-
	Corpus Callosum Body	61	0.1232	0.004458	+	0.0204	-0.02504	-	0.0957	-0.02021	-
	Corpus Callosum Splenium	61	0.2653	0.005416	+	0.0579	-0.02315	-	0.3391	-0.01525	-
	Cortical Spinal Tract Left	57	0.4924	0.002187	+	0.1924	-0.01777	-	0.3063	-0.01924	-
	Cortical Spinal Tract Right	59	0.4552	0.002497	+	0.5342	0.008304	+	0.3391	0.016354	+

	Fronto-occipital Fasciculus Left	54	0.4552	0.001637	+	0.0204	-0.02228	-	**0.0231**	-0.02339	-
	Fronto-occipital Fasciculus Right	53	0.1895	0.004042	+	0.0964	-0.01481	-	0.3391	-0.00811	-
	Inferior Longitudinal Fasciculus Left	56	0.2653	0.003226	+	0.0204	-0.02356	-	0.0957	-0.02028	-
	Inferior Longitudinal Fasciculus Right	55	0.4198	0.002104	+	0.1409	-0.01556	-	0.2563	-0.01459	-
	Superior Longitudinal Fasciculus Left	59	0.2653	0.002769	+	0.0579	-0.02591	-	0.0957	-0.02635	-
	Superior Longitudinal Fasciculus Right	58	0.1232	0.00463	+	0.0204	-0.02101	-	0.0957	-0.01504	-
**APGAR, 5 minutes**	Corpus Callosum Genu	61	0.2904	0.003963	+	0.6875	-0.00555	-	0.8742	0.006575	+
	Corpus Callosum Body	61	0.1634	0.004592	+	0.6875	-0.01251	-	0.8742	-0.00187	-
	Corpus Callosum Splenium	61	0.1634	0.008438	+	0.6875	-0.01086	-	0.8742	0.012254	+
	Cortical Spinal Tract Left	57	0.5029	0.00249	+	0.7035	0.005859	+	0.8742	0.014558	+
	Cortical Spinal Tract Right	59	0.4765	0.002874	+	0.6875	0.009563	+	0.8742	0.020581	+
	Fronto-occipital Fasciculus Left	54	0.3866	0.002599	+	0.7035	-0.00356	-	0.8742	0.004899	+
	Fronto-occipital Fasciculus Right	53	0.1634	0.004869	+	0.1386	-0.02341	-	0.8742	-0.01585	-
	Inferior Longitudinal Fasciculus Left	56	0.1634	0.006061	+	0.6875	-0.01185	-	0.8742	0.00258	+
	Inferior Longitudinal Fasciculus Right	55	0.2904	0.003437	+	0.6875	-0.01318	-	0.8742	-0.00782	-
	Superior Longitudinal Fasciculus Left	59	0.4765	0.001896	+	0.6875	-0.00804	-	0.8742	-0.00441	-
	Superior Longitudinal Fasciculus Right	58	0.4765	0.001941	+	0.6875	-0.00639	-	0.8742	-0.00238	-
**22q11 Microdeletion**	Corpus Callosum Genu	46	0.9096	0.004524	+	0.9906	-0.00056	-	0.961	0.013357	+
	Corpus Callosum Body	46	0.9096	0.007271	+	0.7319	-0.03722	-	0.961	-0.02951	-
	Corpus Callosum Splenium	46	0.7869	0.034045	+	0.7319	-0.10199	-	0.961	-0.04828	-
	Cortical Spinal Tract Left	42	0.941	0.003405	+	0.7319	-0.08227	-	0.961	-0.10025	-
	Cortical Spinal Tract Right	43	0.7869	0.021142	+	0.7319	-0.085	-	0.961	-0.05942	-
	Fronto-occipital Fasciculus Left	38	0.7869	0.012393	+	0.7319	-0.02936	-	0.961	0.006426	+
	Fronto-occipital Fasciculus Right	36	0.7869	0.014518	+	0.7319	-0.06666	-	0.961	-0.05045	-
	Inferior Longitudinal Fasciculus Left	40	0.9096	-0.00631	-	0.7319	-0.0476	-	0.961	-0.07053	-

	Inferior Longitudinal Fasciculus Right	39	0.9096	0.00615	+	0.7319	-0.05518	-	0.961	-0.06086	-
	Superior Longitudinal Fasciculus Left	44	0.941	-0.00092	-	0.9906	-0.00138	-	0.961	-0.01377	-
	Superior Longitudinal Fasciculus Right	42	0.7869	0.016203	+	0.7319	-0.03794	-	0.961	-0.00332	-
**Single Ventricle**	Corpus Callosum Genu	67	0.9614	0.006882	+	0.8841	-0.00586	-	0.8026	0.006035	+
	Corpus Callosum Body	67	0.9614	0.004047	+	0.8559	0.010961	+	0.7147	0.019943	+
	Corpus Callosum Splenium	67	0.9614	0.000578	+	0.8559	0.018196	+	0.7147	0.029212	+
	Cortical Spinal Tract Left	63	0.9614	-0.0045	-	0.5118	0.064438	+	0.4787	0.061966	+
	Cortical Spinal Tract Right	64	0.9614	0.004986	+	0.5118	0.055479	+	0.3256	0.080949	+
	Fronto-occipital Fasciculus Left	59	0.9614	-0.0013	-	0.8559	0.011687	+	0.7361	0.010946	+
	Fronto-occipital Fasciculus Right	57	0.9614	-0.00309	-	0.5118	0.040936	+	0.3256	0.047956	+
	Inferior Longitudinal Fasciculus Left	61	0.9614	-0.00035	-	0.6068	0.030765	+	0.4787	0.040103	+
	Inferior Longitudinal Fasciculus Right	60	0.9614	0.000374	+	0.5118	0.045284	+	0.3256	0.062734	+
	Superior Longitudinal Fasciculus Left	65	0.9614	0.003375	+	0.6068	-0.03648	-	0.4787	-0.04561	-
	Superior Longitudinal Fasciculus Right	63	0.9614	0.007169	+	0.9739	-0.00091	-	0.7361	0.011353	+
**Arch obstruction**	Corpus Callosum Genu	67	0.9723	0.010894	+	0.9876	-0.01335	-	0.9361	0.007534	+
	Corpus Callosum Body	67	0.9723	0.003374	+	0.9876	0.007257	+	0.9361	0.014738	+
	Corpus Callosum Splenium	67	0.9723	0.004031	+	0.9876	0.000536	+	0.9361	0.010751	+
	Cortical Spinal Tract Left	63	0.9723	0.004722	+	0.9876	0.019943	+	0.9361	0.030089	+
	Cortical Spinal Tract Right	64	0.9723	0.000339	+	0.8151	0.07475	+	0.3729	0.100608	+
	Fronto-occipital Fasciculus Left	59	0.9723	-0.00063	-	0.9876	-0.00136	-	0.9361	-0.00443	-
	Fronto-occipital Fasciculus Right	57	0.9723	0.001534	+	0.9876	0.030729	+	0.5419	0.042267	+
	Inferior Longitudinal Fasciculus Left	61	0.9723	0.002565	+	0.9876	-0.00438	-	0.9361	0.0027	+
	Inferior Longitudinal Fasciculus Right	60	0.9723	0.003111	+	0.9876	0.028836	+	0.6399	0.043902	+
	Superior Longitudinal Fasciculus Left	65	0.9723	0.003801	+	0.9876	-0.02272	-	0.9361	-0.02816	-
	Superior Longitudinal Fasciculus Right	63	0.9723	0.004913	+	0.9876	0.020741	+	0.5419	0.03724	+

**Single ventricle with arch obstruction**	Corpus Callosum Genu	67	0.7832	0.010591	+	0.8717	-0.00759	-	0.5606	0.015311	+
	Corpus Callosum Body	67	0.7832	0.00752	+	0.8717	0.007339	+	0.5313	0.026024	+
	Corpus Callosum Splenium	67	0.7921	0.005563	+	0.8717	0.011256	+	0.5313	0.035432	+
	Cortical Spinal Tract Left	63	0.7832	0.014734	+	0.8717	0.012699	+	0.5313	0.045863	+
	Cortical Spinal Tract Right	64	0.7921	0.007969	+	0.8717	0.021902	+	0.5313	0.047329	+
	Fronto-occipital Fasciculus Left	59	0.7921	-0.0046	-	0.8717	0.02496	+	0.5313	0.023111	+
	Fronto-occipital Fasciculus Right	57	0.7921	-0.00369	-	0.8717	0.030712	+	0.5313	0.033573	+
	Inferior Longitudinal Fasciculus Left	61	0.8235	0.002456	+	0.8717	0.008691	+	0.5606	0.02044	+
	Inferior Longitudinal Fasciculus Right	60	0.7921	-0.00429	-	0.8717	0.040004	+	0.5313	0.041784	+
	Superior Longitudinal Fasciculus Left	65	0.9523	0.0004	+	0.8717	-0.02434	-	0.5313	-0.03474	-
	Superior Longitudinal Fasciculus Right	63	0.7832	0.009445	+	0.8717	0.004782	+	0.5313	0.026708	+
**d-TGA**	Corpus Callosum Genu	67	0.7614	-0.01362	-	0.9879	0.027239	+	0.9853	0.005252	+
	Corpus Callosum Body	67	0.7974	-0.00356	-	0.9879	0.002471	+	0.9853	0.000765	+
	Corpus Callosum Splenium	67	0.7614	-0.00955	-	0.9879	-0.02269	-	0.9853	-0.05688	-
	Cortical Spinal Tract Left	63	0.7974	-0.00496	-	0.9879	-0.00395	-	0.9853	-0.01187	-
	Cortical Spinal Tract Right	64	0.7614	-0.00906	-	0.9879	-0.02873	-	0.9853	-0.05973	-
	Fronto-occipital Fasciculus Left	59	0.7974	-0.00233	-	0.9879	-0.01098	-	0.9853	-0.01847	-
	Fronto-occipital Fasciculus Right	57	0.7974	-0.00303	-	0.9879	0.000447	+	0.9853	-0.0086	-
	Inferior Longitudinal Fasciculus Left	61	0.8208	-0.00173	-	0.9879	-0.00849	-	0.9853	-0.01577	-
	Inferior Longitudinal Fasciculus Right	60	0.7614	-0.00602	-	0.9879	-0.0155	-	0.9853	-0.03638	-
	Superior Longitudinal Fasciculus Left	65	0.7614	-0.00706	-	0.9879	0.034258	+	0.9853	0.031794	+
	Superior Longitudinal Fasciculus Right	63	0.7614	-0.01026	-	0.9879	0.017208	+	0.9853	0.000487	+
**Conotruncal defects**	Corpus Callosum Genu	67	0.9852	-0.01244	-	0.8787	0.031486	+	0.854	0.011879	+
	Corpus Callosum Body	67	0.9852	-0.00308	-	0.8787	0.019452	+	0.854	0.019977	+
	Corpus Callosum Splenium	67	0.9852	0.001471	+	0.8787	0.01019	+	0.854	0.01645	+

	Cortical Spinal Tract Left	63	0.9852	0.003437	+	0.8787	-0.01294	-	0.9708	-0.00176	-
	Cortical Spinal Tract Right	64	0.9852	0.005717	+	0.8787	-0.04913	-	0.7532	-0.05548	-
	Fronto-occipital Fasciculus Left	59	0.9852	0.002302	+	0.9559	-0.00143	-	0.9043	0.006015	+
	Fronto-occipital Fasciculus Right	57	0.9852	-0.00014	-	0.8787	-0.03476	-	0.6144	-0.04544	-
	Inferior Longitudinal Fasciculus Left	61	0.9852	-0.00094	-	0.8787	-0.00951	-	0.854	-0.01706	-
	Inferior Longitudinal Fasciculus Right	60	0.9852	-0.0083	-	0.8787	-0.03443	-	0.6144	-0.07029	-
	Superior Longitudinal Fasciculus Left	65	0.9852	-0.00317	-	0.8787	0.025159	+	0.854	0.031026	+
	Superior Longitudinal Fasciculus Right	63	0.9852	-0.00808	-	0.8787	-0.00755	-	0.7532	-0.02956	-
**Altered fetal cerebral substrate delivery (Y/N)**	Corpus Callosum Genu	67	0.6835	-0.01446	-	0.6962	0.024721	+	0.9243	-0.0128	-
	Corpus Callosum Body	67	0.7695	-0.00611	-	0.6962	0.037624	+	0.9243	0.028719	+
	Corpus Callosum Splenium	67	0.6835	-0.02603	-	0.6962	0.063147	+	0.9243	0.007136	+
	Cortical Spinal Tract Left	63	0.7695	0.007459	+	0.6962	0.051032	+	0.9243	0.080463	+
	Cortical Spinal Tract Right	64	0.9879	0.000268	+	0.8842	-0.01316	-	0.9243	-0.02642	-
	Fronto-occipital Fasciculus Left	59	0.7209	-0.0104	-	0.8842	-0.00703	-	0.9243	-0.0482	-
	Fronto-occipital Fasciculus Right	57	0.6835	-0.01447	-	0.6962	0.03634	+	0.9243	0.017342	+
	Inferior Longitudinal Fasciculus Left	61	0.6835	-0.01441	-	0.6962	0.040979	+	0.9243	0.006228	+
	Inferior Longitudinal Fasciculus Right	60	0.6835	-0.02064	-	0.6962	0.06083	+	0.9243	0.02504	+
	Superior Longitudinal Fasciculus Left	65	0.7695	0.004878	+	0.6962	-0.04964	-	0.9243	-0.05584	-
	Superior Longitudinal Fasciculus Right	63	0.7695	0.007622	+	0.6962	-0.05288	-	0.9243	-0.06073	-
**Altered fetal cerebral substrate delivery severity score**	Corpus Callosum Genu	67	0.9947	-0.00874	-	0.9623	0.008013	+	0.7647	-0.01184	-
	Corpus Callosum Body	67	0.9947	0.00116	+	0.9623	-0.01231	-	0.7801	-0.01003	-
	Corpus Callosum Splenium	67	0.9947	-0.01166	-	0.9623	-0.00454	-	0.7647	-0.03694	-
	Cortical Spinal Tract Left	63	0.9947	0.005306	+	0.9623	0.004365	+	0.7647	0.019471	+
	Cortical Spinal Tract Right	64	0.9947	-0.00358	-	0.9623	-0.01429	-	0.7647	-0.03119	-
	Fronto-occipital Fasciculus Left	59	0.9947	-0.00367	-	0.9623	-0.01668	-	0.7469	-0.03374	-

	Fronto-occipital Fasciculus Right	57	0.9947	-0.00244	-	0.9623	-0.00896	-	0.7647	-0.01692	-
	Inferior Longitudinal Fasciculus Left	61	0.9947	-0.00151	-	0.9623	-0.01575	-	0.7647	-0.02683	-
	Inferior Longitudinal Fasciculus Right	60	0.9947	-0.00532	-	0.9623	-0.01239	-	0.7647	-0.03159	-
	Superior Longitudinal Fasciculus Left	65	0.9947	-0.00021	-	0.9623	0.00159	+	0.9177	0.003762	+
	Superior Longitudinal Fasciculus Right	63	0.9947	4.31E-05	+	0.9623	-0.02665	-	0.7469	-0.03371	-
**Heterotaxy**	Corpus Callosum Genu	67	0.6823	0.008076	+	0.9619	0.013758	+	0.9712	0.040155	+
	Corpus Callosum Body	67	0.6823	0.009907	+	0.9619	-0.02381	-	0.9712	-0.01129	-
	Corpus Callosum Splenium	67	0.6823	0.022114	+	0.9619	-0.01577	-	0.9712	0.04345	+
	Cortical Spinal Tract Left	63	0.6823	-0.00729	-	0.9619	0.039912	+	0.9712	0.030931	+
	Cortical Spinal Tract Right	64	0.6823	0.006673	+	0.9619	0.09922	+	0.8602	0.140218	+
	Fronto-occipital Fasciculus Left	59	0.6823	0.008314	+	0.9619	-0.01596	-	0.9712	0.001628	+
	Fronto-occipital Fasciculus Right	57	0.6823	0.007157	+	0.9619	-0.02559	-	0.9712	-0.01525	-
	Inferior Longitudinal Fasciculus Left	61	0.6823	0.006786	+	0.9619	-0.01681	-	0.9712	-0.00758	-
	Inferior Longitudinal Fasciculus Right	60	0.6823	0.010898	+	0.9619	-0.04693	-	0.9712	-0.03357	-
	Superior Longitudinal Fasciculus Left	65	0.6823	-0.0045	-	0.9645	0.003516	+	0.9712	-0.00456	-
	Superior Longitudinal Fasciculus Right	63	0.6823	-0.00554	-	0.9645	0.002066	+	0.9712	-0.01323	-

FDR = false discovery rate, TGA = transposition of the great arteries

Supplement Table 2(B). Correlation between Clinical Risk Factors and Seed-Based Tractography Measurements: Preoperative Factors

			Fractional Anisotropy			Radial Diffusivity			Axial Diffusivity		
**Independent**	Dependent										
**Clinical Factors**	Tract	N used	FDR p-value	estimate	direction	FDR p-value	estimate	direction	FDR p-value	estimate	direction
**Preoperative ABG pH**	Corpus Callosum Genu	49	0.3314	-0.07075	-	0.3314	0.307776	+	0.3314	0.26299	+
	Corpus Callosum Body	49	0.7733	-0.02191	-	0.7733	0.158816	+	0.7733	0.198414	+
	Corpus Callosum Splenium	49	0.7871	-0.00709	-	0.7871	0.150975	+	0.7871	0.209561	+
	Cortical Spinal Tract Left	49	0.7871	0.046419	+	0.7871	-0.30004	-	0.7871	-0.14687	-
	Cortical Spinal Tract Right	48	0.9965	0.033128	+	0.9965	-0.10271	-	0.9965	-0.00181	-
	Fronto-occipital Fasciculus Left	44	0.3601	-0.01471	-	0.3601	0.230523	+	0.3601	0.275436	+
	Fronto-occipital Fasciculus Right	44	0.2457	0.014959	+	0.2457	0.275502	+	0.2457	0.41909	+
	Inferior Longitudinal Fasciculus Left	45	0.8016	-0.00028	-	0.8016	0.073216	+	0.8016	0.09269	+
	Inferior Longitudinal Fasciculus Right	45	0.7871	-0.00569	-	0.7871	0.126899	+	0.7871	0.18291	+
	Superior Longitudinal Fasciculus Left	47	0.2457	-0.0528	-	0.2457	0.345473	+	0.2457	0.37555	+
	Superior Longitudinal Fasciculus Right	47	0.2457	-0.13868	-	0.2457	0.540069	+	0.2457	0.387389	+
**Preoperative arterial blood gas pO2**	Corpus Callosum Genu	46	0.1478	0.000153	+	0.1478	-0.00112	-	0.1478	-0.00112	-
	Corpus Callosum Body	46	0.1478	3.89E-05	+	0.1478	-0.00131	-	0.1478	-0.00171	-
	Corpus Callosum Splenium	46	0.2091	-1.50E-05	-	0.2091	-0.00117	-	0.2091	-0.00169	-
	Cortical Spinal Tract Left	46	0.1839	-0.00021	-	0.1839	-0.00097	-	0.1839	-0.00189	-
	Cortical Spinal Tract Right	45	0.4092	-0.00033	-	0.4092	-0.0002	-	0.4092	-0.00117	-
	Fronto-occipital Fasciculus Left	43	0.1478	-0.00012	-	0.1478	-0.00087	-	0.1478	-0.00145	-
	Fronto-occipital Fasciculus Right	43	0.1478	-9.70E-05	-	0.1478	-0.00084	-	0.1478	-0.00137	-
	Inferior Longitudinal Fasciculus Left	44	0.2091	-0.00013	-	0.2091	-0.00069	-	0.2091	-0.00124	-
	Inferior Longitudinal Fasciculus Right	43	0.2091	-8.70E-06	-	0.2091	-0.00105	-	0.2091	-0.00146	-
	Superior Longitudinal Fasciculus Left	44	0.2091	-2.90E-05	-	0.2091	-0.00065	-	0.2091	-0.00098	-
	Superior Longitudinal Fasciculus Right	44	0.1478	0.00015	+	0.1478	-0.00136	-	0.1478	-0.00143	-
**Preoperative arterial lactate**	Corpus Callosum Genu	61	0.6741	-9.90E-05	-	0.6741	-0.00947	-	0.6741	-0.01387	-
	Corpus Callosum Body	61	0.6741	-0.00063	-	0.6741	-0.01496	-	0.6741	-0.01915	-
	Corpus Callosum Splenium	61	0.6741	-0.01098	-	0.6741	0.005968	+	0.6741	-0.01907	-

	Cortical Spinal Tract Left	59	0.6741	-0.01226	-	0.6741	0.057295	+	0.6741	0.04009	+
	Cortical Spinal Tract Right	59	0.4576	-0.00837	-	0.4576	0.076284	+	0.4576	0.076886	+
	Fronto-occipital Fasciculus Left	55	0.6741	0.003895	+	0.6741	-0.02227	-	0.6741	-0.01945	-
	Fronto-occipital Fasciculus Right	53	0.6741	-0.00355	-	0.6741	0.01411	+	0.6741	0.010941	+
	Inferior Longitudinal Fasciculus Left	56	0.6741	-0.0014	-	0.6741	0.014433	+	0.6741	0.021102	+
	Inferior Longitudinal Fasciculus Right	56	0.6741	-0.00204	-	0.6741	0.012494	+	0.6741	0.017148	+
	Superior Longitudinal Fasciculus Left	59	0.6741	0.002505	+	0.6741	0.017154	+	0.6741	0.028751	+
	Superior Longitudinal Fasciculus Right	57	0.6741	-0.00102	-	0.6741	-0.01863	-	0.6741	-0.0269	-
**Preoperative hepatic dysfunction**	Corpus Callosum Genu	54	0.9615	0.053991	+	0.9615	-0.17228	-	0.9615	-0.08263	-
	Corpus Callosum Body	54	0.9615	0.006071	+	0.9615	-0.06592	-	0.9615	-0.05331	-
	Corpus Callosum Splenium	54	0.9615	0.043953	+	0.9615	-0.04122	-	0.9615	0.095591	+
	Cortical Spinal Tract Left	53	0.9615	0.012144	+	0.9615	-0.18937	-	0.9615	-0.22839	-
	Cortical Spinal Tract Right	53	0.9615	-0.01818	-	0.9615	-0.07387	-	0.9615	-0.15125	-
	Fronto-occipital Fasciculus Left	49	0.9615	0.030854	+	0.9615	-0.06994	-	0.9615	-0.00477	-
	Fronto-occipital Fasciculus Right	48	0.9615	0.017796	+	0.9615	-0.04842	-	0.9615	-0.01221	-
	Inferior Longitudinal Fasciculus Left	50	0.9615	0.017752	+	0.9615	-0.00032	-	0.9615	0.061029	+
	Inferior Longitudinal Fasciculus Right	48	0.9615	0.016081	+	0.9615	-0.05117	-	0.9615	-0.01436	-
	Superior Longitudinal Fasciculus Left	52	0.9615	0.019517	+	0.9615	-0.07513	-	0.9615	-0.04411	-
	Superior Longitudinal Fasciculus Right	52	0.9615	0.015254	+	0.9615	-0.01276	-	0.9615	0.02523	+
**Preoperative inotrope use**	Corpus Callosum Genu	67	0.9331	-0.01753	-	0.9331	0.040097	+	0.9331	0.007437	+
	Corpus Callosum Body	67	0.9331	-0.0203	-	0.9331	0.041737	+	0.9331	0.005419	+
	Corpus Callosum Splenium	67	0.9331	-0.01454	-	0.9331	0.028888	+	0.9331	-0.00738	-
	Cortical Spinal Tract Left	63	0.9331	-0.01745	-	0.9331	0.014416	+	0.9331	-0.0119	-
	Cortical Spinal Tract Right	64	0.9331	0.001277	+	0.9331	-0.01335	-	0.9331	-0.00407	-
	Fronto-occipital Fasciculus Left	59	0.9331	-0.01331	-	0.9331	0.03693	+	0.9331	0.014566	+
	Fronto-occipital Fasciculus Right	57	0.9331	-0.0096	-	0.9331	0.020252	+	0.9331	0.004794	+
	Inferior Longitudinal Fasciculus Left	61	0.3377	-0.01218	-	0.3377	0.061444	+	0.3377	0.054249	+
	Inferior Longitudinal Fasciculus Right	60	0.3377	-0.01561	-	0.3377	0.068817	+	0.3377	0.056773	+

	Superior Longitudinal Fasciculus Left	65	0.3377	-0.0153	-	0.3377	0.096748	+	0.3377	0.086187	+
	Superior Longitudinal Fasciculus Right	63	0.3377	-0.02188	-	0.3377	0.073775	+	0.3377	0.044289	+
**Age at surgery**	Corpus Callosum Genu	67	0.9345	-0.00117	-	0.9345	0.002572	+	0.9345	0.000376	+
	Corpus Callosum Body	67	0.2644	-0.0011	-	0.2644	0.005815	+	0.2644	0.004589	+
	Corpus Callosum Splenium	67	0.3052	-0.00172	-	0.3052	0.007654	+	0.3052	0.004677	+
	Cortical Spinal Tract Left	63	0.9345	0.000569	+	0.9345	-0.00221	-	0.9345	-0.00031	-
	Cortical Spinal Tract Right	64	0.5506	-0.0002	-	0.5506	-0.00273	-	0.5506	-0.00305	-
	Fronto-occipital Fasciculus Left	59	0.0572	-0.00041	-	0.0572	0.005624	+	0.0572	0.005786	+
	Fronto-occipital Fasciculus Right	57	0.3052	-0.00066	-	0.3052	0.003603	+	0.3052	0.002929	+
	Inferior Longitudinal Fasciculus Left	61	0.5506	-0.00076	-	0.5506	0.003818	+	0.5506	0.002213	+
	Inferior Longitudinal Fasciculus Right	60	0.3052	-0.00084	-	0.3052	0.004909	+	0.3052	0.004056	+
	Superior Longitudinal Fasciculus Left	65	0.3052	-0.00074	-	0.3052	0.004375	+	0.3052	0.004525	+
	Superior Longitudinal Fasciculus Right	63	0.0908	-0.00103	-	0.0908	0.005758	+	0.0908	0.004881	+
**Age at surgery ≤ 7 days**	Corpus Callosum Genu	67	0.6552	0.005772	+	0.6552	-0.01686	-	0.6552	-0.01356	-
	Corpus Callosum Body	67	0.6228	0.006861	+	0.6228	-0.0352	-	0.6228	-0.02943	-
	Corpus Callosum Splenium	67	0.6228	0.002637	+	0.6228	-0.03574	-	0.6228	-0.04378	-
	Cortical Spinal Tract Left	63	0.5118	-0.01148	-	0.5118	0.079991	+	0.5118	0.063906	+
	Cortical Spinal Tract Right	64	0.6228	-0.00341	-	0.6228	0.046749	+	0.6228	0.042013	+
	Fronto-occipital Fasciculus Left	59	0.2618	-0.0028	-	0.2618	-0.04199	-	0.2618	-0.06239	-
	Fronto-occipital Fasciculus Right	57	0.4528	-0.00563	-	0.4528	-0.01998	-	0.4528	-0.04586	-
	Inferior Longitudinal Fasciculus Left	61	0.7795	-0.00047	-	0.7795	-0.0086	-	0.7795	-0.00995	-
	Inferior Longitudinal Fasciculus Right	60	0.6228	-0.00084	-	0.6228	-0.01593	-	0.6228	-0.02655	-
	Superior Longitudinal Fasciculus Left	65	0.6228	0.008183	+	0.6228	-0.02897	-	0.6228	-0.02875	-
	Superior Longitudinal Fasciculus Right	63	0.4528	0.012389	+	0.4528	-0.05539	-	0.4528	-0.04582	-
**Post-conceptional age at surgery**	Corpus Callosum Genu	67	0.6785	-0.00774	-	0.6785	0.00561	+	0.6785	-0.01209	-
	Corpus Callosum Body	67	0.6785	-0.00597	-	0.6785	0.020584	+	0.6785	0.011906	+
	Corpus Callosum Splenium	67	0.6785	-0.00888	-	0.6785	0.033556	+	0.6785	0.017444	+
	Cortical Spinal Tract Left	63	0.771	0.002729	+	0.771	0.001417	+	0.771	0.01288	+

	Cortical Spinal Tract Right	64	0.6785	0.000472	+	0.6785	-0.0276	-	0.6785	-0.03094	-
	Fronto-occipital Fasciculus Left	59	0.6785	-0.00053	-	0.6785	0.021217	+	0.6785	0.023276	+
	Fronto-occipital Fasciculus Right	57	0.8559	-0.001	-	0.8559	0.005772	+	0.8559	0.004038	+
	Inferior Longitudinal Fasciculus Left	61	0.9475	-0.00356	-	0.9475	0.010604	+	0.9475	0.001122	+
	Inferior Longitudinal Fasciculus Right	60	0.7794	-0.00452	-	0.7794	0.016032	+	0.7794	0.008978	+
	Superior Longitudinal Fasciculus Left	65	0.6785	-0.00133	-	0.6785	0.012333	+	0.6785	0.016586	+
	Superior Longitudinal Fasciculus Right	63	0.6785	-0.00299	-	0.6785	0.013194	+	0.6785	0.009479	+

ABG = arterial blood gas, FDR = false discovery rate

Supplemental Table 2(C): Correlation between Clinical Risk Factors and Seed-Based Tractography Measurements: Intra-operative Factors

Independent	Dependent		Fractional Anisotropy			Radial Diffusivity			Axial Diffusivity		
**Clinical Factors**	Tract	N used	FDR p-value	estimate	direction	FDR p-value	estimate	direction	FDR p-value	estimate	direction
**Cardiopulmonary bypass used**	Corpus Callosum Genu	77	0.735	0.016777	+	0.735	-0.0159	-	0.735	0.028427	+
	Corpus Callosum Body	76	0.4484	-2.60E-05	-	0.4484	0.050815	+	0.4484	0.074409	+
	Corpus Callosum Splenium	77	0.1441	0.011002	+	0.1441	0.083436	+	0.1441	0.159482	+
	Cortical Spinal Tract Left	64	0.735	0.021301	+	0.735	-0.06349	-	0.735	-0.03302	-
	Cortical Spinal Tract Right	68	0.1558	0.008048	+	0.1558	-0.10109	-	0.1558	-0.10565	-
	Fronto-occipital Fasciculus Left	66	0.1558	0.01027	+	0.1558	0.039807	+	0.1558	0.081429	+
	Fronto-occipital Fasciculus Right	68	0.1558	0.008341	+	0.1558	0.062665	+	0.1558	0.103124	+
	Inferior Longitudinal Fasciculus Left	70	0.9028	0.000844	+	0.9028	-0.00328	-	0.9028	0.006017	+
	Inferior Longitudinal Fasciculus Right	70	0.1558	0.006367	+	0.1558	0.041095	+	0.1558	0.078695	+
	Superior Longitudinal Fasciculus Left	72	0.8049	0.008416	+	0.8049	0.000371	+	0.8049	0.018732	+
	Superior Longitudinal Fasciculus Right	75	0.735	-0.00806	-	0.735	0.033651	+	0.735	0.024155	+
**Cardiopulmonary bypass time**	Corpus Callosum Genu	68	0.2055	-0.00028	-	0.2055	0.000868	+	0.2055	0.000506	+
	Corpus Callosum Body	68	0.546	-0.00011	-	0.546	0.00042	+	0.546	0.000339	+
	Corpus Callosum Splenium	68	0.0875	-0.00024	-	0.0875	0.00107	+	0.0875	0.000952	+
	Cortical Spinal Tract Left	56	0.3848	3.66E-05	+	0.3848	0.000175	+	0.3848	0.000414	+
	Cortical Spinal Tract Right	60	0.7093	3.64E-05	+	0.7093	2.42E-05	+	0.7093	0.000169	+
	Fronto-occipital Fasciculus Left	57	**0.0242**	-9.30E-05	-	**0.0242**	0.000793	+	**0.0242**	0.000821	+
	Fronto-occipital Fasciculus Right	59	0.099	-5.90E-05	-	0.099	0.00062	+	0.099	0.000711	+
	Inferior Longitudinal Fasciculus Left	61	0.1493	-7.80E-05	-	0.1493	0.000585	+	0.1493	0.000602	+
	Inferior Longitudinal Fasciculus Right	61	0.2224	-6.60E-05	-	0.2224	0.000392	+	0.2224	0.000431	+
	Superior Longitudinal Fasciculus Left	64	0.8158	-1.10E-05	-	0.8158	9.54E-05	+	0.8158	0.000108	+
	Superior Longitudinal Fasciculus Right	66	0.9628	-9.00E-05	-	0.9628	0.000143	+	0.9628	1.24E-05	+
**Aortic cross-clamp used**	Corpus Callosum Genu	73	0.9332	-0.01579	-	0.9332	0.034647	+	0.9332	0.018625	+
	Corpus Callosum Body	72	0.9332	-0.01097	-	0.9332	0.01802	+	0.9332	0.004487	+
	Corpus Callosum Splenium	73	0.6977	-0.02547	-	0.6977	0.075579	+	0.6977	0.050983	+
	Cortical Spinal Tract Left	60	0.9332	-0.0055	-	0.9332	0.013854	+	0.9332	0.012673	+
	Cortical Spinal Tract Right	64	0.766	-0.00062	-	0.766	-0.03389	-	0.766	-0.03616	-
	Fronto-occipital Fasciculus Left	62	0.0688	-0.01318	-	0.0688	0.079158	+	0.0688	0.074776	+
	Fronto-occipital Fasciculus Right	64	0.1368	-0.00989	-	0.1368	0.064878	+	0.1368	0.072317	+
	Inferior Longitudinal Fasciculus Left	66	0.9332	-0.01443	-	0.9332	0.019871	+	0.9332	-0.0029	-
	Inferior Longitudinal Fasciculus Right	66	0.0688	-0.01159	-	0.0688	0.071165	+	0.0688	0.078215	+
	Superior Longitudinal Fasciculus Left	68	0.9332	-0.00628	-	0.9332	0.018914	+	0.9332	0.010478	+
	Superior Longitudinal Fasciculus Right	71	0.9332	-0.02162	-	0.9332	0.039107	+	0.9332	0.006731	+
**Aortic cross-clamp time**	Corpus Callosum Genu	50	**0.0033**	-0.00027	-	**0.0033**	0.001807	+	**0.0033**	0.001756	+
	Corpus Callosum Body	50	0.3738	2.86E-05	+	0.3738	0.000765	+	0.3738	0.000955	+
	Corpus Callosum Splenium	50	**0.0033**	-0.00022	-	**0.0033**	0.002161	+	**0.0033**	0.002439	+
	Cortical Spinal Tract Left	40	0.4586	0.000134	+	0.4586	0.000132	+	0.4586	0.000518	+
	Cortical Spinal Tract Right	43	0.4586	0.00016	+	0.4586	-2.10E-05	-	0.4586	0.000357	+
	Fronto-occipital Fasciculus Left	42	0.3738	-2.40E-05	-	0.3738	0.000549	+	0.3738	0.000582	+
	Fronto-occipital Fasciculus Right	42	0.3738	4.94E-05	+	0.3738	0.000613	+	0.3738	0.000909	+

	Inferior Longitudinal Fasciculus Left	44	0.3738	1.66E-05	+	0.3738	0.000503	+	0.3738	0.000718	+
	Inferior Longitudinal Fasciculus Right	43	0.5436	-1.90E-05	-	0.5436	0.00023	+	0.5436	0.000332	+
	Superior Longitudinal Fasciculus Left	48	0.7624	0.000141	+	0.7624	-3.40E-05	-	0.7624	0.000177	+
	Superior Longitudinal Fasciculus Right	49	0.5436	0.000141	+	0.5436	5.47E-05	+	0.5436	0.000326	+
**Circulatory Arrest/ DHCA used**	Corpus Callosum Genu	74	0.8783	0.002428	+	0.8783	-0.00525	-	0.8783	-0.00144	-
	Corpus Callosum Body	73	0.9062	0.002194	+	0.9062	-0.00443	-	0.9062	-0.00053	-
	Corpus Callosum Splenium	74	0.8783	0.000956	+	0.8783	-0.00337	-	0.8783	-0.00293	-
	Cortical Spinal Tract Left	61	0.8783	0.001633	+	0.8783	-0.00384	-	0.8783	-0.00173	-
	Cortical Spinal Tract Right	65	0.8783	0.001175	+	0.8783	-0.00402	-	0.8783	-0.00305	-
	Fronto-occipital Fasciculus Left	63	0.8783	0.001372	+	0.8783	-0.00409	-	0.8783	-0.00226	-
	Fronto-occipital Fasciculus Right	65	0.8783	0.001525	+	0.8783	-0.00507	-	0.8783	-0.00419	-
	Inferior Longitudinal Fasciculus Left	67	0.8783	0.001105	+	0.8783	-0.00332	-	0.8783	-0.00191	-
	Inferior Longitudinal Fasciculus Right	67	0.9062	0.001415	+	0.9062	-0.00182	-	0.9062	0.000412	+
	Superior Longitudinal Fasciculus Left	69	0.8783	0.001224	+	0.8783	-0.00014	-	0.8783	0.002729	+
	Superior Longitudinal Fasciculus Right	72	0.8783	0.001809	+	0.8783	-0.00371	-	0.8783	-0.00107	-
**Circulatory Arrest/ DHCA time**	Corpus Callosum Genu	63	0.5665	0.000627	+	0.5665	-0.0024	-	0.5665	-0.00186	-
	Corpus Callosum Body	63	0.9711	0.000227	+	0.9711	-0.00052	-	0.9711	-4.60E-05	-
	Corpus Callosum Splenium	63	0.9711	0.00098	+	0.9711	-0.00176	-	0.9711	0.000125	+
	Cortical Spinal Tract Left	51	0.9711	0.000652	+	0.9711	-0.00102	-	0.9711	-0.00017	-
	Cortical Spinal Tract Right	56	0.9711	0.000401	+	0.9711	-0.00047	-	0.9711	-4.90E-05	-
	Fronto-occipital Fasciculus Left	52	0.662	0.000366	+	0.662	-0.00132	-	0.662	-0.0009	-
	Fronto-occipital Fasciculus Right	54	0.6899	0.000216	+	0.6899	0.000389	+	0.6899	0.000908	+
	Inferior Longitudinal Fasciculus Left	57	0.8211	0.000269	+	0.8211	-0.00096	-	0.8211	-0.00061	-
	Inferior Longitudinal Fasciculus 1(a) Right	56	0.662	0.000293	+	0.662	-0.00115	-	0.662	-0.00104	-
	Superior Longitudinal Fasciculus Left	59	0.662	-8.50E-05	-	0.662	-0.00119	-	0.662	-0.00158	-
	Superior Longitudinal Fasciculus Right	61	0.662	0.000356	+	0.662	-0.00135	-	0.662	-0.00089	-

DHCA = deep hypothermic circulatory arrest, FDR = false discovery rate

Supplemental Table 2(D). Correlation between Clinical Risk Factors and Seed-Based Tractography Measurements: Post-operative Factors

Independent	Dependent		Fractional Anisotropy			Radial Diffusivity			Axial Diffusivity		
Clinical Factors	Tract	N used	FDR p-value	estimate	direction	FDR p-value	estimate	direction	FDR p-value	estimate	Direction
ECMO during 1st hospitalization	Corpus Callosum Genu	76	0.7058	-0.03412	-	0.7058	0.029078	+	0.7058	-0.05894	-
	Corpus Callosum Body	75	0.9448	-0.03663	-	0.9448	0.073901	+	0.9448	-0.004	-
	Corpus Callosum Splenium	76	0.9448	-0.055	-	0.9448	0.114426	+	0.9448	0.007934	+
	Cortical Spinal Tract Left	63	0.7058	-0.03556	-	0.7058	0.022746	+	0.7058	-0.05494	-
	Cortical Spinal Tract Right	67	0.9448	-0.01327	-	0.9448	0.011401	+	0.9448	-0.01411	-
	Fronto-occipital Fasciculus Left	65	0.752	-0.03055	-	0.752	0.083559	+	0.752	0.027344	+
	Fronto-occipital Fasciculus Right	67	0.7058	-0.0199	-	0.7058	0.074555	+	0.7058	0.041133	+
	Inferior Longitudinal Fasciculus Left	69	0.752	-0.02982	-	0.752	0.079034	+	0.752	0.028892	+
	Inferior Longitudinal Fasciculus Right	69	0.7058	-0.02244	-	0.7058	0.09357	+	0.7058	0.067048	+
	Superior Longitudinal Fasciculus Left	71	0.7058	-0.02308	-	0.7058	0.071616	+	0.7058	0.042119	+
	Superior Longitudinal Fasciculus Right	74	0.7058	-0.02336	-	0.7058	0.003069	+	0.7058	-0.04522	-
Time on ECMO	Corpus Callosum Genu	74	0.9759	-0.00184	-	0.9759	0.007313	+	0.9759	0.001751	+
	Corpus Callosum Body	73	0.9759	-0.00329	-	0.9759	0.012814	+	0.9759	0.004724	+
	Corpus Callosum Splenium	74	0.8118	-0.00833	-	0.8118	0.031195	+	0.8118	0.019203	+
	Cortical Spinal Tract Left	61	0.8118	-0.00149	-	0.8118	-0.01004	-	0.8118	-0.01942	-
	Cortical Spinal Tract Right	65	0.8118	0.001005	+	0.8118	-0.01054	-	0.8118	-0.013	-
	Fronto-occipital Fasciculus Left	63	0.9759	0.000188	+	0.9759	0.002638	+	0.9759	0.001247	+
	Fronto-occipital Fasciculus Right	65	0.9759	0.002573	+	0.9759	-0.00074	-	0.9759	0.000345	+
	Inferior Longitudinal Fasciculus Left	67	0.8461	-0.00083	-	0.8461	0.007676	+	0.8461	0.007593	+
	Inferior Longitudinal Fasciculus Right	67	0.8461	0.000773	+	0.8461	0.003993	+	0.8461	0.005959	+
	Superior Longitudinal Fasciculus Left	69	0.8118	0.000795	+	0.8118	0.007455	+	0.8118	0.011451	+
	Superior Longitudinal Fasciculus Right	72	0.8118	0.002414	+	0.8118	-0.0097	-	0.8118	-0.00836	-
Delayed sternal closure	Corpus Callosum Genu	77	0.4591	0.001451	+	0.4591	0.015924	+	0.4591	0.037505	+
	Corpus Callosum Body	76	0.4075	-0.00391	-	0.4075	0.043925	+	0.4075	0.057448	+
	Corpus Callosum Splenium	77	0.2387	0.004541	+	0.2387	0.063487	+	0.2387	0.117711	+
	Cortical Spinal Tract Left	64	0.6958	-0.01221	-	0.6958	0.000317	+	0.6958	-0.0236	-

	Cortical Spinal Tract Right	68	0.4075	-0.01277	-	0.4075	-0.02809	-	0.4075	-0.05528	-
	Fronto-occipital Fasciculus Left	66	0.3586	-0.00057	-	0.3586	0.038475	+	0.3586	0.055234	+
	Fronto-occipital Fasciculus Right	68	0.3586	0.006578	+	0.3586	0.02644	+	0.3586	0.056496	+
	Inferior Longitudinal Fasciculus Left	70	0.6958	0.001908	+	0.6958	-0.02591	-	0.6958	-0.01924	-
	Inferior Longitudinal Fasciculus Right	70	0.3586	0.00918	+	0.3586	0.025376	+	0.3586	0.06232	+
	Superior Longitudinal Fasciculus Left	72	0.3586	0.004687	+	0.3586	0.035092	+	0.3586	0.059167	+
	Superior Longitudinal Fasciculus Right	75	0.8872	-0.00204	-	0.8872	0.003901	+	0.8872	0.004807	+
**Had unplanned intervention(s) during 1st hospitalization**	Corpus Callosum Genu	76	0.8511	-0.00813	-	0.8511	0.00778	+	0.8511	-0.01236	-
	Corpus Callosum Body	75	0.8511	-0.01276	-	0.8511	0.028277	+	0.8511	0.005469	+
	Corpus Callosum Splenium	76	0.964	-0.01261	-	0.964	0.022702	+	0.964	0.000815	+
	Cortical Spinal Tract Left	63	0.8511	-0.01074	-	0.8511	0.00863	+	0.8511	-0.01285	-
	Cortical Spinal Tract Right	67	0.8511	-0.00191	-	0.8511	-0.00133	-	0.8511	-0.00511	-
	Fronto-occipital Fasciculus Left	65	0.8511	-0.00427	-	0.8511	0.013027	+	0.8511	0.004533	+
	Fronto-occipital Fasciculus Right	67	0.8511	0.000111	+	0.8511	0.017955	+	0.8511	0.020675	+
	Inferior Longitudinal Fasciculus Left	69	0.8511	-0.00243	-	0.8511	0.009397	+	0.8511	0.006882	+
	Inferior Longitudinal Fasciculus Right	69	0.8511	-0.00281	-	0.8511	0.010306	+	0.8511	0.006863	+
	Superior Longitudinal Fasciculus Left	71	0.8511	-0.00247	-	0.8511	0.007263	+	0.8511	0.005677	+
	Superior Longitudinal Fasciculus Right	74	0.7887	-0.00464	-	0.7887	-0.00975	-	0.7887	-0.02081	-
**ICU length of stay, 1st hospitalization**	Corpus Callosum Genu	77	0.1133	-0.0002	-	0.1133	0.001343	+	0.1133	0.001081	+
	Corpus Callosum Body	76	0.0682	-0.00032	-	0.0682	0.002049	+	0.0682	0.001635	+
	Corpus Callosum Splenium	77	0.0682	-0.00028	-	0.0682	0.001826	+	0.0682	0.001774	+
	Cortical Spinal Tract Left	64	0.4908	-0.00015	-	0.4908	-0.0002	-	0.4908	-0.0007	-
	Cortical Spinal Tract Right	68	0.3999	6.12E-06	+	0.3999	0.000657	+	0.3999	0.000854	+
	Fronto-occipital Fasciculus Left	66	0.6714	-0.00017	-	0.6714	0.000194	+	0.6714	-0.00022	-
	Fronto-occipital Fasciculus Right	68	0.1262	-7.40E-05	-	0.1262	0.001064	+	0.1262	0.001079	+
	Inferior Longitudinal Fasciculus Left	70	0.5452	8.46E-05	+	0.5452	0.000167	+	0.5452	0.00045	+
	Inferior Longitudinal Fasciculus Right	70	0.3056	-0.00014	-	0.3056	-0.00019	-	0.3056	-0.00068	-
	Superior Longitudinal Fasciculus Left	72	0.5153	-8.40E-05	-	0.5153	0.000519	+	0.5153	0.000463	+

	Superior Longitudinal Fasciculus Right	75	0.5153	-4.80E-05	-	0.5153	0.000378	+	0.5153	0.000354	+
**Hospital length of stay (days)**	Corpus Callosum Genu	77	0.1991	-0.00017	-	0.1991	0.001045	+	0.1991	0.000766	+
	Corpus Callosum Body	76	0.0677	-0.00027	-	0.0677	0.001768	+	0.0677	0.001455	+
	Corpus Callosum Splenium	77	0.0677	-0.00023	-	0.0677	0.001493	+	0.0677	0.001418	+
	Cortical Spinal Tract Left	64	0.5042	-9.60E-05	-	0.5042	-9.70E-05	-	0.5042	-0.00047	-
	Cortical Spinal Tract Right	68	0.4444	9.33E-06	+	0.4444	0.000477	+	0.4444	0.000632	+
	Fronto-occipital Fasciculus Left	66	0.5042	-0.00013	-	0.5042	2.64E-05	+	0.5042	-0.00035	-
	Fronto-occipital Fasciculus Right	68	0.0913	-9.30E-05	-	0.0913	0.001053	+	0.0913	0.001019	+
	Inferior Longitudinal Fasciculus Left	70	0.896	-3.70E-05	-	0.896	9.79E-05	+	0.896	6.57E-05	+
	Inferior Longitudinal Fasciculus Right	70	0.407	-0.00013	-	0.407	-6.10E-05	-	0.407	-0.00051	-
	Superior Longitudinal Fasciculus Left	72	0.5042	-8.10E-05	-	0.5042	0.000473	+	0.5042	0.000404	+
	Superior Longitudinal Fasciculus Right	75	0.7669	-2.30E-06	-	0.7669	0.000155	+	0.7669	0.000145	+
**Expired during 1st hospitalization**	Corpus Callosum Genu	77	0.7469	-0.06229	-	0.7469	0.102809	+	0.7469	-0.04365	-
	Corpus Callosum Body	76	0.6814	-0.06301	-	0.6814	0.068919	+	0.6814	-0.08296	-
	Corpus Callosum Splenium	77	0.8863	-0.04778	-	0.8863	0.082769	+	0.8863	-0.01361	-
	Cortical Spinal Tract Left	64	0.7469	0.014382	+	0.7469	-0.06153	-	0.7469	-0.05604	-
	Cortical Spinal Tract Right	68	0.5766	-0.01471	-	0.5766	0.114571	+	0.5766	0.125818	+
	Fronto-occipital Fasciculus Left	66	0.7469	-0.06108	-	0.7469	0.150587	+	0.7469	0.049177	+
	Fronto-occipital Fasciculus Right	68	0.0902	-0.02612	-	0.0902	0.212878	+	0.0902	0.21198	+
	Inferior Longitudinal Fasciculus Left	70	0.2382	0.024092	+	0.2382	0.072168	+	0.2382	0.166984	+
	Inferior Longitudinal Fasciculus Right	70	0.7469	-0.03041	-	0.7469	0.045295	+	0.7469	-0.02939	-
	Superior Longitudinal Fasciculus Left	72	0.2382	-0.02791	-	0.2382	-0.05479	-	0.2382	-0.13449	-
	Superior Longitudinal Fasciculus Right	75	0.132	-0.04177	-	0.132	-0.04552	-	0.132	-0.13627	-
	Corpus Callosum Genu	77	0.2805	-0.04129	-	0.2805	0.002462	+	0.2805	-0.1258	-
	Corpus Callosum Body	76	0.951	-0.05284	-	0.951	0.079766	+	0.951	-0.03755	-
	Corpus Callosum Splenium	77	0.4092	-0.06263	-	0.4092	0.025056	+	0.4092	-0.13809	-
	Cortical Spinal Tract Left	64	0.951	-0.03756	-	0.951	0.048189	+	0.951	-0.01711	-
	Cortical Spinal Tract Right	68	0.951	-0.02054	-	0.951	0.045018	+	0.951	0.013218	+

**Required CPR, 1st hospitalization**	Fronto-occipital Fasciculus Left	66	0.951	-0.04951	-	0.951	0.069117	+	0.951	-0.03622	-
	Fronto-occipital Fasciculus Right	68	0.951	-0.02077	-	0.951	0.0366	+	0.951	-0.00455	-
	Inferior Longitudinal Fasciculus Left	70	0.951	-0.00909	-	0.951	-0.02814	-	0.951	-0.07204	-
	Inferior Longitudinal Fasciculus Right	70	0.951	-0.02903	-	0.951	0.035669	+	0.951	-0.01976	-
	Superior Longitudinal Fasciculus Left	72	0.951	-0.0193	-	0.951	0.051894	+	0.951	0.024812	+
	Superior Longitudinal Fasciculus Right	75	0.0682	-0.03126	-	0.0682	-0.07255	-	0.0682	-0.15072	-
**Seizures, 1st hospitalization**	Corpus Callosum Genu	77	0.928	-0.01981	-	0.928	0.040585	+	0.928	-0.00791	-
	Corpus Callosum Body	76	0.928	-0.02357	-	0.928	0.048572	+	0.928	-0.00779	-
	Corpus Callosum Splenium	77	0.928	-0.02338	-	0.928	0.055041	+	0.928	0.011654	+
	Cortical Spinal Tract Left	64	0.8362	0.009867	+	0.8362	-0.05503	-	0.8362	-0.05355	-
	Cortical Spinal Tract Right	68	0.928	-0.01524	-	0.928	0.031432	+	0.928	0.004684	+
	Fronto-occipital Fasciculus Left	66	0.928	-0.00111	-	0.928	0.001407	+	0.928	-0.0055	-
	Fronto-occipital Fasciculus Right	68	0.8362	-0.00383	-	0.8362	0.046043	+	0.8362	0.035399	+
	Inferior Longitudinal Fasciculus Left	70	0.8362	-0.01104	-	0.8362	0.059667	+	0.8362	0.050683	+
	Inferior Longitudinal Fasciculus Right	70	0.8362	-0.01471	-	0.8362	-0.00618	-	0.8362	-0.04778	-
	Superior Longitudinal Fasciculus Left	72	0.8362	-0.00598	-	0.8362	-0.0159	-	0.8362	-0.0365	-
	Superior Longitudinal Fasciculus Right	75	0.8362	-0.0127	-	0.8362	-0.01584	-	0.8362	-0.04335	-
**Discharged on antiepileptics**	Corpus Callosum Genu	77	0.8885	-0.01866	-	0.8885	0.052081	+	0.8885	0.011252	+
	Corpus Callosum Body	76	0.513	-0.02364	-	0.513	0.112211	+	0.513	0.073857	+
	Corpus Callosum Splenium	77	0.4396	-0.03409	-	0.4396	0.13782	+	0.4396	0.092712	+
	Cortical Spinal Tract Left	64	0.8885	-0.01934	-	0.8885	0.021952	+	0.8885	-0.01226	-
	Cortical Spinal Tract Right	68	0.9169	-0.02445	-	0.9169	0.045799	+	0.9169	0.00526	+
	Fronto-occipital Fasciculus Left	66	0.7713	-0.01594	-	0.7713	0.055985	+	0.7713	0.028876	+
	Fronto-occipital Fasciculus Right	68	0.8885	-0.0158	-	0.8885	0.045901	+	0.8885	0.013148	+
	Inferior Longitudinal Fasciculus Left	70	0.4396	-0.02475	-	0.4396	0.103357	+	0.4396	0.070695	+
	Inferior Longitudinal Fasciculus Right	70	0.513	-0.0217	-	0.513	0.07475	+	0.513	0.047209	+
	Superior Longitudinal Fasciculus Left	72	0.2904	-0.01649	-	0.2904	0.111209	+	0.2904	0.105435	+
	Superior Longitudinal Fasciculus Right	75	0.7713	-0.01789	-	0.7713	0.05664	+	0.7713	0.030899	+

**Discharged with gastrostomy tube**	Corpus Callosum Genu	77	0.9463	-0.02378	-	0.9463	0.045061	+	0.9463	-0.00656	-
	Corpus Callosum Body	76	0.9463	-0.03185	-	0.9463	0.097851	+	0.9463	0.038822	+
	Corpus Callosum Splenium	77	0.9463	-0.03991	-	0.9463	0.112127	+	0.9463	0.047781	+
	Cortical Spinal Tract Left	64	0.9463	-0.04113	-	0.9463	0.059039	+	0.9463	-0.01516	-
	Cortical Spinal Tract Right	68	0.1012	-0.02868	-	0.1012	0.138911	+	0.1012	0.112626	+
	Fronto-occipital Fasciculus Left	66	0.9463	-0.01624	-	0.9463	0.01324	+	0.9463	-0.02054	-
	Fronto-occipital Fasciculus Right	68	0.9463	-0.01524	-	0.9463	0.019664	+	0.9463	-0.01074	-
	Inferior Longitudinal Fasciculus Left	70	0.9463	-0.01816	-	0.9463	0.046625	+	0.9463	0.014156	+
	Inferior Longitudinal Fasciculus Right	70	0.9463	-0.0179	-	0.9463	0.045762	+	0.9463	0.017189	+
	Superior Longitudinal Fasciculus Left	72	0.4675	-0.01286	-	0.4675	0.076576	+	0.4675	0.068868	+
	Superior Longitudinal Fasciculus Right	75	0.9472	-0.01614	-	0.9472	0.031585	+	0.9472	0.002139	+
**Discharged with tracheostomy and/or ventilator**	Corpus Callosum Genu	77	0.9107	-0.03908	-	0.9107	0.10018	+	0.9107	0.030602	+
	Corpus Callosum Body	76	0.9107	-0.04677	-	0.9107	0.079856	+	0.9107	-0.02494	-
	Corpus Callosum Splenium	77	0.9107	-0.04274	-	0.9107	0.048099	+	0.9107	-0.05716	-
	Cortical Spinal Tract Left	64	0.9107	-0.02706	-	0.9107	-0.0088	-	0.9107	-0.07655	-
	Cortical Spinal Tract Right	68	0.9107	-0.01912	-	0.9107	0.069307	+	0.9107	0.049811	+
	Fronto-occipital Fasciculus Left	66	0.9107	-0.02801	-	0.9107	0.042716	+	0.9107	-0.00969	-
	Fronto-occipital Fasciculus Right	68	0.9107	-0.04766	-	0.9107	0.016829	+	0.9107	-0.07814	-
	Inferior Longitudinal Fasciculus Left	70	0.9107	-0.04943	-	0.9107	0.100073	+	0.9107	0.030753	+
	Inferior Longitudinal Fasciculus Right	70	0.9107	-0.02542	-	0.9107	0.031502	+	0.9107	-0.0208	-
	Superior Longitudinal Fasciculus Left	72	0.9107	-0.02493	-	0.9107	0.056929	+	0.9107	0.010572	+
	Superior Longitudinal Fasciculus Right	75	0.9107	-0.01791	-	0.9107	0.01787	+	0.9107	-0.02822	-

FDR = false discovery rate, CPR=cardiopulmonary resuscitation, ECMO=extra corporeal membrane oxygenation, ICU=intensive care unit
